# Prognostic Significance of Combination of Preoperative Platelet Count and Neutrophil-Lymphocyte Ratio (COP-NLR) in Patients with Non-Small Cell Lung Cancer: Based on a Large Cohort Study

**DOI:** 10.1371/journal.pone.0126496

**Published:** 2015-05-07

**Authors:** Hua Zhang, Lianmin Zhang, Kaikai Zhu, Bowen Shi, Yuesong Yin, Jinfang Zhu, Dongsheng Yue, Bin Zhang, Changli Wang

**Affiliations:** 1 Department of Lung Cancer, Tianjin Medical University Cancer Institute and Hospital, National Clinical Research Center for Cancer, Tianjin, China; 2 Key Laboratory of Cancer Prevention and Therapy, Tianjin, China; 3 Tianjin Lung Cancer Center, Tianjin, China; 4 Department of Pediatrics, Tianjin Medical University General Hospital, Tianjin, China; Memorial Sloan-Kettering Cancer Center, UNITED STATES

## Abstract

**Introduction:**

The aim of this study was to investigate the prognostic significance of the combination of the preoperative platelet count and neutrophil-lymphocyte ratio (COP-NLR) for predicting postoperative survival of patients undergoing complete resection for non-small cell lung cancer (NSCLC).

**Methods:**

The preoperative COP-NLR was calculated on the basis of data obtained.Patients with both an increased platelet count (>30.0×10^4^ mm^-3^) and an elevated NLR (>2.3) were assigned a score of 2, and patients with one or neither were assigned as a score of 1 or 0, respectively.

**Results:**

A total of 1238 NSCLC patients were enrolled in this analysis. Multivariate analysis using the 15 clinicolaboratory variables selected by univariate analyses demonstrated that the preoperative COP-NLR was an independent prognostic factor for DFS (HR: 1.834, 95%CI: 1.536 to 2.200, *P*<0.001) and OS (HR: 1.810, 95%CI: 1.587 to 2.056, *P*<0.001). In sub-analyses by tumor stage (I, II, IIIA), a significant association was found between DFS and OS and level of COP-NLR in each subgroup (*P*<0.001, *P*=0.002, *P*<0.001 for DFS, respectively; *P*<0.001, *P*=0.001, *P*<0.001 for OS). When the subgroup of patients with high-risk COP-NLR (score of 2) was analyzed, no benefit of adjuvant chemotherapy could be found (*P*=0.237 for DFS and *P*=0.165 for OS).

**Conclusions:**

The preoperative COP-NLR is able to predict the prognosis of patients with NSCLC and divide these patients into three independent groups before surgery. Our results also demonstrate that high-risk patients based on the COP-NLR do not benefit from adjuvant chemotherapy. Independent validation of our findings is warranted.

## Introduction

Lung cancer is the most common cause of cancer-related death worldwide, with 5-year survival rates of less than 15% [1]. Non-small-cell lung cancer (NSCLC) accounts for approximately 80% of all lung cancers. Recently, a large number of prognostic predictors have been reported in the literature for patients with NSCLC, however, the majority of these factors cannot be obtained preoperatively. Moreover, some of these factors are available only as research tools.

Recently, there has been a growing interest in the host inflammatory response to tumors, and systemic inflammatory response has been shown to reflect the promotion of angiogenesis, DNA damage and tumor invasion through up-regulation of cytokines [2–4]. Based on this, a number of inflammation-based prognostic markers have been identified, such as the Glasgow Prognostic Score (GPS) and platelet to lymphocyte ratio (PLR) [5, 6]. In addition, there is increasing evidence that the neutrophil to lymphocyte ratio (NLR) and thrombocytosis can be used for prognostication in lung cancer [7–10]. Recently, two studies investigated a novel inflammation-based prognostic system named the combination of platelet count and NLR (COP-NLR). They revealed that COP-NLR was a useful predictor of postoperative survival in patients with colorectal cancer and gastric cancer [11, 12]. However, to the best of our knowledge, no studies regarding COP-NLR in patients with NSCLC are available. Therefore, in this study, we evaluated the clinical utility of a novel inflammation-based prognostic system (COP-NLR) in patients with NSCLC undergoing complete resection.

## Patients and Methods

### Patients

A retrospective study of patients withNSCLC undergoing surgery was conducted at the Tianjin Medical University Cancer Institute and Hospital from January 2005 to December 2009. The study was approved by the Institutional Review Board of Tianjin Medical University Cancer Institute and Hospital. All patients provided written informed consent. The inclusion criteria were (1) complete pulmonary resection and systematic node dissection of the hilar and mediastinal lymph nodes and (2) histologically confirmed NSCLC. Patients who met the following criteria were excluded from the study: (1) preoperative chemotherapy or radiotherapy; (2) positive surgical margins; (3) advanced disease (e.g., malignant pleural effusion/involvement or distant metastasis); (4) clinical evidence of infection or other bone marrow, hematological or autoimmune disease; (5) a history of lung cancer or a second primary cancer diagnosed within 5 years of the lung cancer index; and (6) patients who died within 1 month after surgery. Based on the inclusion and exclusion criteria, a total of 1238 NSCLC patients were analyzed in the present study. All of the patients were Chinese. The preoperative evaluation included a detailed clinical history and physical examination with a series of biochemistry tests, complete blood cell counts and coagulation tests. Further investigations included radiography, flexible bronchoscopy, chest and upper abdominal computed tomography (CT), radionuclide bone scan, and CT or magnetic resonance imaging (MRI) of the brain. Tumor stages were based on the 7th edition of the TNM Classification [13]. The main adjuvant treatment that patients underwent after operation was chemotherapy or radiotherapy, either alone or in combination. The chemotherapy was routine program for NSCLC, including vinorelbine, paclitaxel, or gemcitabine plus carboplatin or cisplatin.

### Definition of COP-NLR and other variables

Venous blood sampling was taken in a week prior to surgery, and collected in ethylenediaminetetraacetic acid (EDTA) containing tube. NLR was defined as follows: NLR = percentage of neutrophils for whole white blood cells (WBC)/percentage of lymphocytes for whole WBC or NLR = peripheral neutrophil count/peripheral lymphocyte count. The cut-off values of the preoperative platelet count and NLR were decided using receiver operating characteristic (ROC) curve analyses. For the 1238 NSCLC patients in our study, an NLR of 2.3 corresponded to the maximum joint sensitivity and specificity on the ROC plot. The area under the curve (AUC) for NLR was 0.650. Thus, the recommended cut-off value for NLR was 2.3. Similarly, the prominent point on the ROC curve indicated a cut-off value of 29.0 ×10^4^ mm^-3^ for the platelet count,and the area under the ROC curve was 0.562. Therefore, the recommended cut-off value of preoperative platelet count was defined as 30.0 ×10^4^ mm^-3^.

The COP-NLR was calculated on the basis of data obtained. Patients with both an increased platelet count (>30×10^4^ mm^-3^) and an elevated NLR (>2.3) were assigned a score of 2, and patients with one or neither were assigned as a score of 1 or 0, respectively.

### Statistical analysis

All statistical analyses were carried out using SPSS 13.0 software (SPSS Inc., Chicago, USA). Overall survival (OS) was defined as the interval between the date of surgery and the date of death or last follow-up. Disease-free survival (DFS) was defined as the duration of time between the date of surgery and the date of first recurrence or last follow-up. Data are presented as the mean ± s.d. The differences between the three COP-NLR groups and the clinicolaboratory variables were analyzed by chi-square test and Kruskal-Wallis test. Hazard ratios (HR) and 95% confidence intervals (95% CI) were calculated by univariate and multivariate analysis using the Cox proportional hazards model.

Twenty-three clinicolaboratory variables, including age, sex, smoking status, resection type, histological subtype, tumor location, lesion, TNM stage, adjuvant chemotherapy, adjuvant radiotherapy, WBC count, platelet count, neutrophil ratio, lymphocyte ratio, monocyte ratio, NLR, albumin, intraoperative blood loss, hemoglobin (Hb), lactate dehydrogenase (LDH), fibrinogen, D-dimer, and COP-NLR were entered in the univariate analysis. In order to classify the patients into two groups, the cut-off values of clinicolaboratory variables were determined using ROC curve analyses. The value of the maximum joint sensitivity and specificity on the ROC plot was defined as the recommended cut-off value. Clinicolaboratory variables with a *P*-value<0.05 were further analyzed in the multivariate analysis to determine the independent predictors of DFS and OS. The Kaplan-Meier method and the log-rank test were used to compare the survival curves of the three COP-NLR groups.

## Results

Based on the inclusion and exclusion, ultimately, a total of 1238 patients with histologically confirmed NSCLC were enrolled in this study. There were 584 patients with COP-NLR = 0, 488 patients with COP-NLR = 1 and 166 patients with COP-NLR = 2. Of these, 426 (34.4%) were women, and 812 (65.6%) were men. The median age was 60 years, with an age range from 24 to 82 years. The distribution of pathological stages was as follows: stage I, 536; stage II, 264; and stage IIIA, 438. The follow-up period ranged from 2 month to 96 months (median, 45.0 months; mean, 44.1 months). At the end of the last follow up, 686 patients had died. The 5-year OS rate was 47.3% for the whole study population.

The distribution of the clinical background characteristics of the studied patients in the three groups is shown in Table 1. As shown in Table 1, significant differences between COP-NLR and age (*P* = 0.002), sex (*P*<0.001), smoking status (*P* = 0.004), resection type (*P*<0.001), histological subtype (*P*<0.001), lesion (*P*<0.001), and TNM stage (I/II/IIIA) (*P*<0.001) were demonstrated.

**Table 1:** Correlations between COP-NLR and clinical characteristics.

Variables	COP-NLR = 0 (n = 584)	COP-NLR = 1 (n = 488)	COP-NLR = 2 (n = 166)	*P* value
**Age**				
** ≤60**	298(44.8%)	258 (38.7%)	110 (16.5%)	
** >60**	286(50%)	230 (40.2%)	56 (8.8%)	0.002
**Sex**				
** Female**	228 (53.5%)	136 (31.9%)	62 (14.6%)	
** Male**	356 (43.9%)	352 (43.3%)	104 (12.8%)	<0.001
**Smoking status**				
** Yes**	360 (44.6%)	346 (42.8%)	102 (12.6%)	
** No**	224 (52.1%)	142 (33.0%)	64 (14.9%)	0.004
**Resection type**				
** Pneumonectomy**	38 (25.7%)	76 (51.4%)	34 (22.9%)	
** Lobectomy**	546 (50.1%)	412 (37.8%)	132 (12.1%)	<0.001
**Histological subtype**				
** SqCC**	222 (38.1%)	264 (45.4%)	96 (16.5%)	
** Adenocarcinoma**	292 (58.2%)	158 (31.5%)	52 (10.3%)	
** Others**	70 (45.5%)	66 (42.8%)	18 (11.7%)	<0.001
**Tumor location**				
** Left**	250 (49.4%)	200 (39.5%)	56 (11.1%)	
** Right**	334 (45.6%)	288 (39.4%)	110 (15.0%)	0.110
**Lesion**				
** Peripheral**	464 (52.1%)	320 (40.0%)	106 (11.9%)	
** Central**	120 (34.5%)	168 (48.3%)	60 (17.2%)	<0.001
**lymph node metastasis**				
** Yes**	240 (43.8%)	230 (42.0%)	78 (14.2%)	
** No**	344 (49.9%)	258 (37.4%)	88 (12.7%)	0.105
**TNM stage**				
** I**	312 (58.2%)	180 (33.6%)	44 (8.2%)	
** II**	88 (33.3%)	116 (44.0%)	60 (22.7%)	
** IIIA**	184 (42.0%)	192 (43.8%)	62 (14.2%)	<0.001

Abbreviations: Squamous cell carcinoma = SqCC;COP-NLR = combination of preoperative platelet count and neutrophil-lymphocyte ratio.

The relationships between COP-NLR and clinicolaboratory varibles are shown in Table 2. Significant differences between COP-NLR and age (*P*<0.001), maximum tumor diameter (*P*<0.001), WBC count (*P*<0.001), platelet count (*P*<0.001), neutrophil ratio (*P*<0.001), lymphocyte ratio (*P*<0.001), monocyte ratio (*P* = 0.028), Hb (*P*<0.001), LDH (*P* = 0.039), albumin (*P*<0.001), fibrinogen (*P*<0.001), D-dimer (*P* = 0.001), NLR (*P*<0.001), intraoperative blood loss (*P* = 0.030), and survival period (*P*<0.001) were shown.

**Table 2:** Correlations between COP-NLR and clinicolaboratory characteristics.

Variables	COP-NLR = 0 (n = 584)	COP-NLR = 1 (n = 488)	COP-NLR = 2 (n = 166)	*P* value
**Age (year)**	60.9±9.4	60.5±9.2	57.4±9.1	<0.001
**Maximum tumor diameter (cm)**	3.6±1.5	4.7±2.2	5.6±2.7	<0.001
**WBC count (× 10** ^**3**^ **mm** ^**-3**^ **)**	6.3±1.7	7.3±1.7	7.3±1.5	<0.001
**Platelet count (× 10** ^**4**^ **mm** ^**-3**^ **)**	21.8±4.5	26.0±7.8	33.4±6.2	<0.001
**Neutrophil ratio (%)**	54.9±7.5	62.6±10.4	66.0±11.8	<0.001
**Lymphocyte ratio (%)**	33.6±6.3	28.1±2.7	25.0±7.9	<0.001
**Monocyte ratio (%)**	7.7±2.3	8.0±2.5	8.1±2.7	0.028
**Hb (gL** ^**-1**^ **)**	140.7±14.8	139.9±16.6	131.1±14.5	<0.001
**LDH (UL** ^**-1**^ **)**	177.9±39.1	185.2±55.8	177.5±40.3	0.039
**Albumin (gdl** ^**-1**^ **)**	4.4±0.4	4.3±0.4	4.1±0.5	<0.001
**Fibrinogen (gL** ^**-1**^ **)**	3.3±0.8	3.9±1.0	4.2±1.1	<0.001
**D-dimer (mgL** ^**-1**^ **)**	0.18±0.18	0.21±0.27	0.22±0.12	0.001
**NLR**	1.7±0.4	2.7±1.1	3.0±0.7	<0.001
**Intraoperative blood loss (ml)**	182.4±104.2	216.5±231.1	216.0±184.7	0.030
**Survival period (months)**	49.2±23.4	41.0±26.2	36.0±26.0	<0.001

Abbreviations: COP-NLR = combination of preoperative platelet count and neutrophil-lymphocyte ratio; WBC = white blood cell; Hb = hemoglobin; LDH = lactate dehydrogenase; NLR = neutrophil/lymphocyte ratio.

Based on the cut-off value, we separated the patients into different groups. Survival analyses in relation to NLR and platelet count and patient prognosis were performed. In the univariate analysis of OS, NLR and platelet countwere both found to be significant factors, with hazard ratio (HR) = 1.533 (95% confidence interval [CI]: 1.458 to 1.785) for NLR, and HR = 1.379 (95% CI: 1.166 to 1.634) for platelet count. To identify the optimal factor for patient prognosis, we evaluated the prognostic value of COP-NLR. The HR for COP-NLR was 1.813 (95% CI: 1.650 to 2.106), implying a more important prognostic value. For DFS, the HR for COP-NLR was 1.835 (95% CI: 1.580 to 2.132) (Table 3). Other identified prognostic factors for DFS and OS included resection type, lesion, TNM stage, adjuvant radiotherapy, WBC count, neutrophil ratio, lymphocyte ratio, monocyte ratio, albumin, intraoperative blood loss, Hb, LDH, fibrinogen, and D-dimer.

**Table 3:** Univariate analysis of DFS and OS for all NSCLC patients.

		DFS			OS	
	*P* value	HR	95% CI	*P* value	HR	95% CI
**Age (≤60, >60)**	0.677	1.032	0.889 to 1.199	0.492	1.054	0.907 to 1.224
**Sex (female, male)**	0.472	1.059	0.905 to 1.240	0.949	1.005	0.859 to 1.176
**Smoking status (yes, no)**	0.675	0.967	0.825 to 1.132	0.177	0.897	0.765 to 1.051
**Resection type (pneumonectomy, lobectomy)**	0.015	1.307	1.054 to 1.662	0.015	1.308	1.054 to 1.623
**Histological subtype (squamous, adenocarcinoma, others)**	0.700	1.022	0.916 to 1.140	0.872	1.009	0.904 to 1.126
**Tumor location (left, right)**	0.719	0.973	0.836 to 1.132	0.643	0.965	0.829 to 1.123
**Lesion (peripheral, central)**	0.007	1.248	1.062 to 1.467	0.005	1.264	1.075 to 1.486
**TNM stage (I, II, IIIA)**	<0.001	1.801	1.649 to 1.968	<0.001	1.789	1.638 to 1.954
**Adjuvant chemotherapy (yes, no)**	0.278	1.087	0.935 to 1.263	0.414	1.065	0.916 to 1.238
**Adjuvant radiotherapy (yes, no)**	<0.001	1.746	1.427 to 2.137	<0.001	1.574	1.287 to 1.926
**WBC count (× 10** ^**3**^ **mm** ^**-3**^ **)**	0.001	1.311	1.118 to 1.537	0.001	1.300	1.108 to 1.524
**Platelet count (× 10** ^**4**^ **mm** ^**-3**^ **)**	<0.001	1.377	1.164 to 1.631	<0.001	1.379	1.166 to 1.634
**Neutrophil ratio (%)**	<0.001	1.314	1.130 to 1.528	0.001	1.295	1.114 to 1.506
**Lymphocyte ratio (%)**	<0.001	0.659	0.563 to 0.772	<0.001	0.660	0.563 to 0.773
**Monocyte ratio (%)**	<0.001	1.358	1.162 to 1.586	<0.001	1.384	1.185 to 1.617
**NLR**	<0.001	1.509	1.355 to 1.745	<0.001	1.533	1.458 to 1.785
**Albumin (gdl** ^**-1**^ **)**	<0.001	0.670	0.566 to 0.792	<0.001	0.689	0.583 to 0.815
**Intraoperative blood loss (ml)**	0.003	1.258	1.079 to 1.466	0.007	1.235	1.060 to 1.440
**Hb (gL** ^**-1**^ **)**	<0.001	0.666	0.566 to 0.784	<0.001	0.679	0.577 to 0.799
**LDH (UL** ^**-1**^ **)**	<0.001	1.999	1.561 to 2.561	<0.001	1.851	1.445 to 2.371
**Fibrinogen (gL** ^**-1**^ **)**	<0.001	1.335	1.149 to 1.550	0.002	1.370	1.180 to 1.592
**D-dimer (mgL** ^**-1**^ **)**	<0.001	1.415	1.217 to 1.645	<0.001	1.323	1.137 to 1.538
**COP-NLR (0,1,2)**	<0.001	1.835	1.580 to 2.132	<0.001	1.813	1.650 to 2.106

Abbreviations: DFS = disease-free survival; OS = overall survival; HR = hazard ratio; CI = confidence interval; WBC = white blood cell; Hb = hemoglobin; LDH = lactate dehydrogenase; NLR = neutrophil/lymphocyte ratio; COP-NLR = combination of preoperative platelet count and neutrophil-lymphocyte ratio.

Multivariate analysis using the 15 clinicopathological characteristics selected above (excluding platelet count and NLR) demonstrated that preoperative COP-NLR was associated with DFS and OS (HR: 1.834, 95%CI: 1.536 to 2.200, *P*<0.001 and HR: 1.810, 95%CI: 1.587 to 2.056, *P*<0.001, respectively) along with TNM stage, LDH level, and D-dimer (Table 4). At the same time, multivariate analysis also demonstrated that COP-NLR remained an independent prognostic marker in SqCC or adenocarcinoma (Tables 5–8).

**Table 4:** Multivariate analysis of DFS and OS for all NSCLC patients.

		DFS			OS	
	*P* value	HR	95% CI	*P* value	HR	95% CI
**Resection type (pneumonectomy, lobectomy)**	0.940	1.105	0.783 to 1.289	0.952	0.987	0.765 to 1.268
**Lesion (peripheral, central)**	0.568	1.055	0.868 to 1.285	0.851	1.025	0.832 to 1.240
**TNM stage (I, II, IIIA)**	<0.001	1.630	1.473 to 1.812	<0.001	1.658	1.523 to 1.841
**Adjuvant radiotherapy (yes, no)**	0.057	1.341	0.976 to 1.651	0.065	1.236	0.978 to 1.491
**WBC count (× 10** ^**3**^ **mm** ^**-3**^ **)**	0.175	1.136	0.945 to 1.370	0.350	1.085	0.912 to 1.310
**Neutrophil ratio (%)**	0.105	1.330	0.955 to 1.628	0.110	1.329	0.930 to 1.665
**Lymphocyte ratio (%)**	0.155	0.812	0.653 to 1.107	0.110	0.841	0.673 to 1.052
**Monocyte ratio (%)**	0.090	1.190	0.908 to 1.420	0.112	1.245	0.945 to 1.469
**Albumin (gdl** ^**-1**^ **)**	0.063	0.812	0.663 to 1.098	0.070	0.823	0.671 to 1.131
**Intraoperative blood loss (ml)**	0.283	1.100	0.932 to 1.295	0.376	1.082	0.934 to 1.256
**Hb (gL** ^**-1**^ **)**	0.101	0.864	0.721 to 1.035	0.095	0.857	0.713 to 1.045
**LDH (UL** ^**-1**^ **)**	0.001	1.441	1.333 to 1.780	0.002	1.536	1.361 to 1.834
**Fibrinogen (gL** ^**-1**^ **)**	0.135	0.871	0.730 to 1.053	0.410	0.935	0.778 to 1.119
**D-dimer (mgL** ^**-1**^ **)**	0.002	1.289	1.073 to 1.512	0.006	1.258	1.066 to 1.481
**COP-NLR (0,1,2)**	<0.001	1.834	1.536 to 2.200	<0.001	1.810	1.587 to 2.056

Abbreviations: DFS = disease-free survival; OS = overall survival; HR = hazard ratio; CI = confidence interval; WBC = white blood cell; Hb = hemoglobin; LDH = lactate dehydrogenase; COP-NLR = combination of preoperative platelet count and neutrophil-lymphocyte ratio.

**Table 5:** Univariate analysis of DFS and OS for SqCC patients.

		DFS			OS	
	*P* value	HR	95% CI	*P* value	HR	95% CI
**Age (≤60, >60)**	0.115	1.192	0.958 to 1.483	0.100	1.201	0.965 to 1.493
**Sex (female, male)**	0.522	1.100	0.821 to 1.474	0.459	1.117	0.834 to 1.496
**Smoking status (yes, no)**	0.795	1.042	0.766 to 1.416	0.855	0.972	0.714 to 1.323
**Resection type (pneumonectomy, lobectomy)**	0.173	1.202	0.923 to 1.566	0.212	1.183	0.908 to 1.542
**Tumor location (left, right)**	0.694	1.046	0.837 to 1.306	0.745	1.037	0.831 to 1.295
**Lesion (peripheral, central)**	0.131	1.184	0.951 to 1.474	0.096	1.205	0.967 to 1.500
**TNM stage (I, II, IIIA)**	<0.001	1.790	1.568 to 2.042	<0.001	1.761	1.542 to 2.011
**Adjuvant chemotherapy (yes, no)**	0.141	1.180	0.947 to 1.470	0.227	1.145	0.919 to 1.426
**Adjuvant radiotherapy (yes, no)**	<0.001	2.041	1.561 to 2.669	<0.001	1.819	1.392 to 2.376
**WBC count (× 10** ^**3**^ **mm** ^**-3**^ **)**	0.025	1.244	1.015 to 1.554	0.029	1.258	1.067 to 1.484
**Platelet count(× 10** ^**4**^ **mm** ^**-3**^ **)**	0.015	1.346	1.059 to 1.706	0.019	1.330	1.074 to 1.686
**Neutrophil ratio (%)**	0.016	1.210	1.073 to 1.505	0.018	1.254	1.028 to 1.437
**Lymphocyte ratio (%)**	0.001	0.650	0.502to 0.840	0.006	0.696	0.538 to 0.901
**Monocyte ratio (%)**	<0.001	1.605	1.288 to 1.999	<0.001	1.607	1.290 to 2.002
**NLR**	<0.001	1.459	1.274 to 1.659	<0.001	1.463	1.253 to 1.662
**Albumin(gdl** ^**-1**^ **)**	0.002	0.653	0.498 to 0.855	0.004	0.674	0.514 to 0.885
**Intraoperative blood loss (ml)**	0.002	1.427	1.136 to 1.793	0.006	1.380	1.098 to 1.773
**Hb (gL** ^**-1**^ **)**	<0.001	0.581	0.457 to 0.737	<0.001	0.585	0.461 to 0.743
**LDH (UL** ^**-1**^ **)**	<0.001	1.785	1.313 to 2.427	0.002	1.608	1.183 to 2.186
**Fibrinogen (gL** ^**-1**^ **)**	0.007	1.367	1.088 to 1.717	0.009	1.358	1.081 to 1.706
**D-dimer (mgL** ^**-1**^ **)**	<0.001	1.681	1.343 to 2.104	<0.001	1.582	1.264 to 1.981
**COP-NLR (0,1,2)**	<0.001	1.604	1.382 to 1.861	<0.001	1.567	1.349 to 1.819

Abbreviations: DFS = disease-free survival; OS = overall survival; HR = hazard ratio; CI = confidence interval; WBC = white blood cell; Hb = hemoglobin; LDH = lactate dehydrogenase; NLR = neutrophil/lymphocyte ratio; COP-NLR = combination of preoperative platelet count and neutrophil-lymphocyte ratio.

**Table 6:** Multivariate analysis of DFS and OS for SqCC patients.

		DFS			OS	
	*P* value	HR	95% CI	*P* value	HR	95% CI
**TNM stage (I, II, IIIA)**	<0.001	1.677	1.426 to 1.972	<0.001	1.723	1.463 to 2.028
**Adjuvant radiotherapy (yes, no)**	0.121	1.343	0.982 to 1.618	0.115	1.378	0.988 to 1.597
**WBC count (× 10** ^**3**^ **mm** ^**-3**^ **)**	0.219	1.169	0.911 to 1.501	0.269	1.097	0.853 to 1.410
**Neutrophil ratio (%)**	0.295	1.064	0.778 to1.456	0.305	1.078	0.792 to 1.468
**Lymphocyte ratio (%)**	0.318	0.835	0.586 to 1.190	0.293	0.876	0.673 to 1.095
**Monocyte ratio (%)**	0.055	1.378	0.965 to 1.782	0.079	1.361	0.972 to 1.755
**Albumin(g dl** ^**-1**^ **)**	0.074	0.750	0.546 to 1.028	0.092	0.759	0.551 to 1.046
**Intraoperative blood loss (ml)**	0.950	1.008	0.779 to 1.304	0.426	1.045	0.750 to 1.258
**Hb (gL** ^**-1**^ **)**	0.520	0.910	0.683 to 1.213	0.326	0.889	0.665 to 1.188
**LDH (UL** ^**-1**^ **)**	<0.001	1.620	1.430 to 2.045	0.010	1.540	1.109 to 2.138
**Fibrinogen (gL** ^**-1**^ **)**	0.056	0.758	0.578 to 1.015	0.077	0.782	0.595 to 1.027
**D-dimer (mgL** ^**-1**^ **)**	0.001	1.528	1.194 to 1.957	0.001	1.512	1.183 to 1.934
**COP-NLR (0,1,2)**	<0.001	1.776	1.433 to 2.012	<0.001	1.735	1.434 to 2.005

Abbreviations: DFS = disease-free survival; OS = overall survival; HR = hazard ratio; CI = confidence interval; WBC = white blood cell; Hb = haemoglobin; LDH = lactate dehydrogenase; COP-NLR = combination of preoperative platelet count and neutrophil-lymphocyte ratio.

**Table 7:** Univariate analysis of DFS and OS for adenocarcinoma patients.

		DFS			OS	
	*P* value	HR	95% CI	*P* value	HR	95% CI
**Age (≤60, >60)**	0.785	0.967	0.761 to 1.229	0.928	1.011	0.796 to 1.284
**Sex (female, male)**	0.679	1.052	0.828 to 1.335	0.871	0.980	0.772 to 1.245
**Smoking status (yes, no)**	0.027	0.765	0.604 to 0.970	0.003	0.699	0.552 to 0.886
**Resection type (pneumonectomy, lobectomy)**	0.437	1.222	0.737 to 2.027	0.328	1.288	0.776 to 2.139
**Tumor location (left, right)**	0.728	1.044	0.819 to 1.330	0.764	1.038	0.815 to 1.322
**Lesion (peripheral, central)**	0.232	1.217	0.882 to 1.681	0.258	1.206	0.872 to 1.667
**TNM stage (I, II, IIIA)**	<0.001	1.694	1.485 to 1.934	<0.001	1.683	1.475 to 1.920
**Adjuvant chemotherapy (yes, no)**	0.481	0.919	0.725 to 1.163	0.305	0.884	0.698 to 1.119
**Adjuvant radiotherapy (yes, no)**	0.019	1.475	1.041 to 2.090	0.012	1.362	0.962 to 1.930
**WBC count (× 10** ^**3**^ **mm** ^**-3**^ **)**	0.003	1.517	1.153 to 1.995	<0.001	1.686	1.281 to 2.219
**Platelet count(× 10** ^**4**^ **mm** ^**-3**^ **)**	0.017	1.406	1.064 to 1.862	0.012	1.430	1.082 to 1.894
**Neutrophil ratio (%)**	0.005	1.419	1.108 to 1.817	0.003	1.462	1.142 to 1.871
**Lymphocyte ratio (%)**	<0.001	0.651	0.513 to 0.825	<0.001	0.624	0.492 to 0.791
**Monocyte ratio (%)**	0.037	1.264	1.005 to 1.513	0.024	1.311	1.112 to 1.612
**NLR**	<0.001	1.731	1.519 to 2.103	<0.001	1.756	1.580 to 2.053
**Albumin(gdl** ^**-1**^ **)**	<0.001	0.628	0.489 to 0.805	0.001	0.646	0.503 to 0.830
**Intraoperative blood loss (ml)**	0.043	1.247	1.057 to 1.455	0.037	1.196	1.015 to 1.440
**Hb (gL** ^**-1**^ **)**	0.002	0.675	0.525 to 0.867	0.004	0.694	0.540 to 0.891
**LDH (UL** ^**-1**^ **)**	<0.001	2.603	1.611 to 4.205	<0.001	2.862	1.769 to 4.630
**Fibrinogen (gL** ^**-1**^ **)**	<0.001	1.592	1.243 to 2.039	<0.001	1.673	1.305 to 2.145
**D-dimer (mgL** ^**-1**^ **)**	0.026	1.311	1.033 to 1.664	0.034	1.266	1.006 to 1.610
**COP-NLR (0,1,2)**	<0.001	1.769	1.542 to 2.035	<0.001	1.807	1.582 to 2.093

Abbreviations: DFS = disease-free survival; OS = overall survival; HR = hazard ratio; CI = confidence interval; WBC = white blood cell; Hb = haemoglobin; LDH = lactate dehydrogenase; NLR = neutrophil/lymphocyte ratio; COP-NLR = combination of preoperative platelet count and neutrophil-lymphocyte ratio.

**Table 8:** Multivariate analysis of DFS and OS for adenocarcinoma patients.

		DFS			OS	
	*P* value	HR	95% CI	*P* value	HR	95% CI
**Smoking status (yes, no)**	0.003	0.659	0.500 to 0.867	<0.001	0.598	0.453 to 0.790
**TNM stage (I, II, IIIA)**	<0.001	1.465	1.265 to 1.697	<0.001	1.499	1.295 to 1.735
**Adjuvant radiotherapy (yes, no)**	0.143	1.309	0.913 to 1.877	0.308	1.206	0.841 to 1.730
**WBC count (× 10** ^**3**^ **mm** ^**-3**^ **)**	0.365	1.162	0.840 to 1.607	0.138	1.282	0.923 to 1.780
**Neutrophil ratio (%)**	0.064	1.115	0.883 to 1.450	0.073	1.121	0.923 to 1.503
**Lymphocyte ratio (%)**	0.057	0.900	0.615 to 1.237	0.065	0.873	0.596 to 1.189
**Monocyte ratio (%)**	0.352	1.119	0.835 to 1.500	0.414	1.131	0.841 to 1.521
**Albumin(gdl** ^**-1**^ **)**	0.009	0.669	0.496 to 0.904	0.010	0.671	0.496 to 0.908
**Intraoperative blood loss (ml)**	0.122	1.242	0.944 to 1.633	0.145	1.227	0.932 to 1.616
**Hb (gL** ^**-1**^ **)**	0.001	0.603	0.448 to 0.813	<0.001	0.569	0.422 to 0.768
**LDH (UL** ^**-1**^ **)**	0.076	1.746	0.944 to 3.231	0.063	1.926	0.996 to 3.616
**Fibrinogen (gL** ^**-1**^ **)**	0.091	1.285	0.961 to 1.718	0.061	1.350	0.973 to 1.800
**D-dimer (mgL** ^**-1**^ **)**	0.234	1.179	0.899 to 1.546	0.253	1.175	0.892 to 1.548
**COP-NLR (0,1,2)**	<0.001	1.858	1.506 to 2.205	<0.001	1.871	1.497 to 2.346

Abbreviations: DFS = disease-free survival; OS = overall survival; HR = hazard ratio; CI = confidence interval; WBC = white blood cell; Hb = haemoglobin; LDH = lactate dehydrogenase; COP-NLR = combination of preoperative platelet count and neutrophil-lymphocyte ratio.

**Fig 1:**
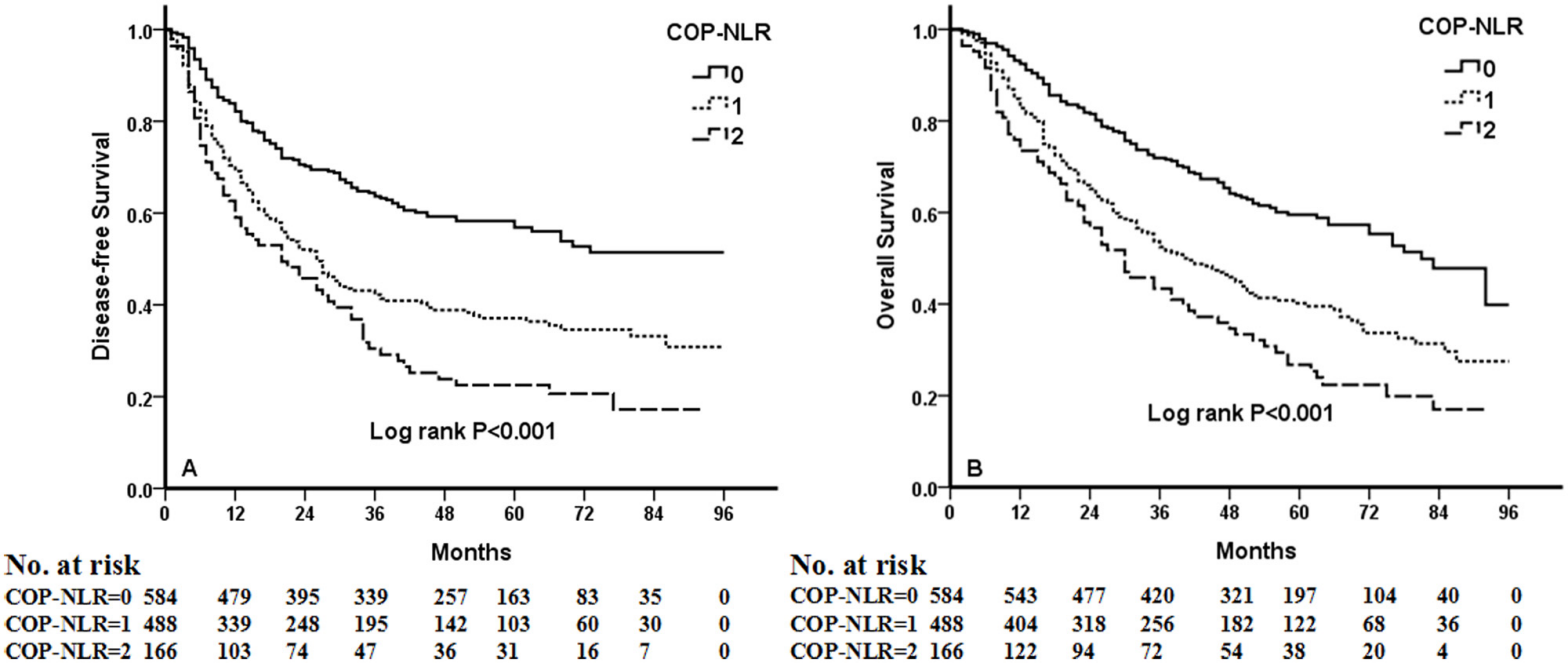
Kaplan-Meier curves for 1238 NSCLC patients. (A) Kaplan-Meier curve of DFS for NSCLC. (B) Kaplan-Meier curve of OS for NSCLC.

**Fig 2:**
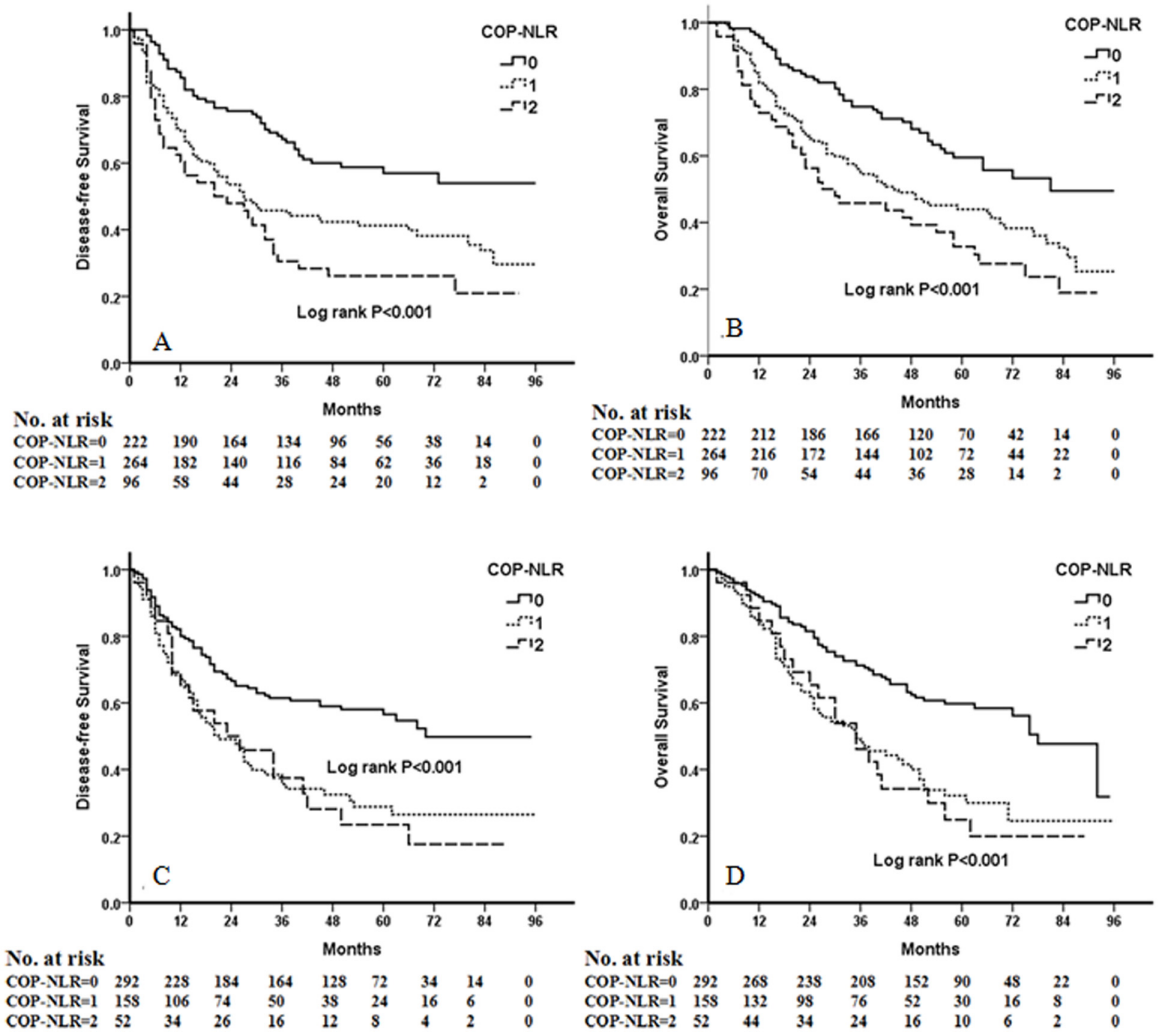
Kaplan-Meiercurves for SqCC patients andadenocarcinoma patients. (A) Kaplan-Meier curve of DFS for SqCC. (B) Kaplan-Meier curve of OS for SqCC. (C) Kaplan-Meier curve of DFS for adenocarcinoma. (D) Kaplan-Meier curve of OS for adenocarcinoma.

**Fig 3:**
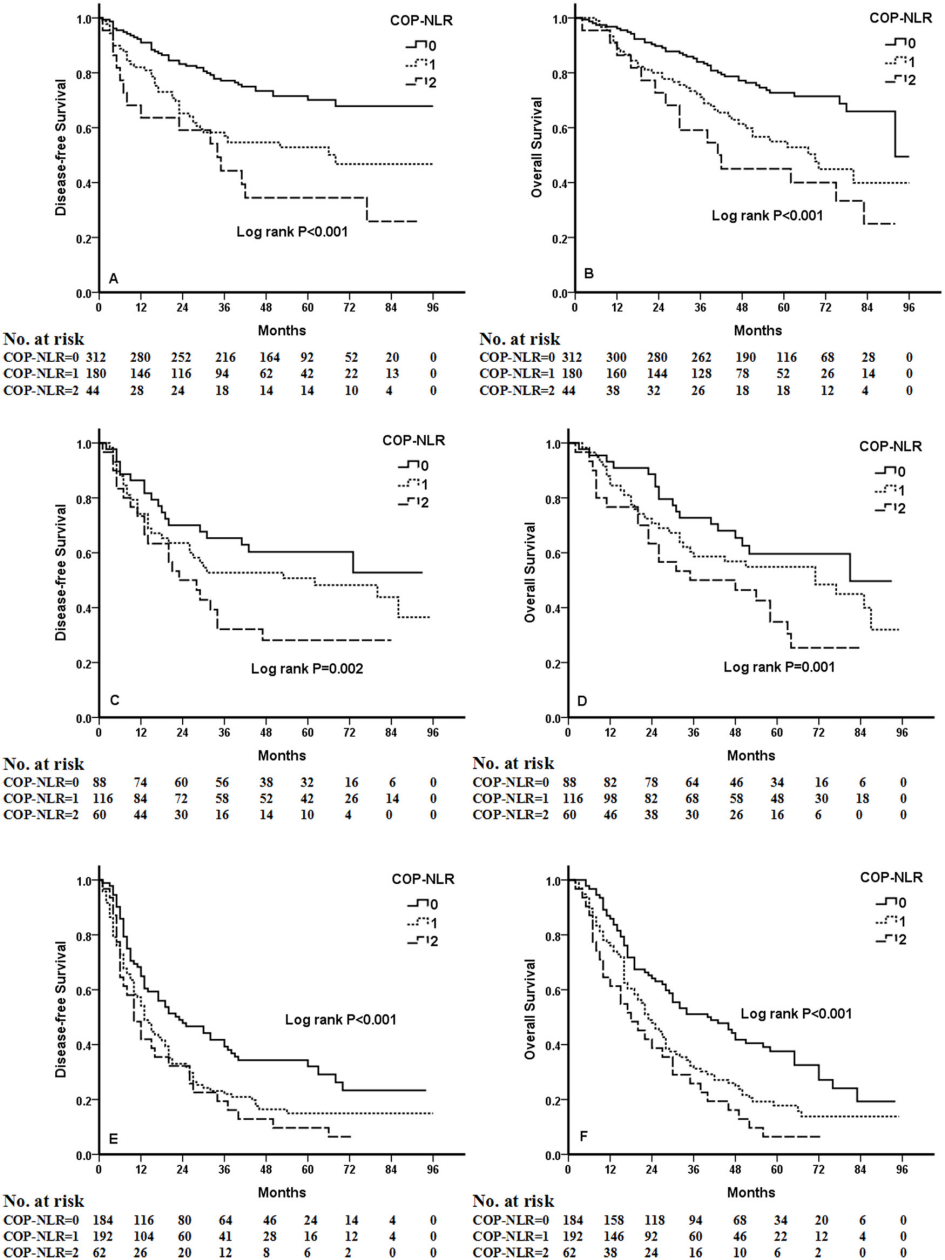
Kaplan-Meier curves of stage I, II and IIIA NSCLC patients. (A) Kaplan-Meier curve of DFS for stage I. (B) Kaplan-Meier curve of OS for stage I. (C) Kaplan-Meier curve of DFS for stage II. (D) Kaplan-Meier curve of OS for stage II. (E) Kaplan-Meier curve of DFS for stage IIIA. (F) Kaplan-Meier curve of OS for stage IIIA.

**Fig 4:**
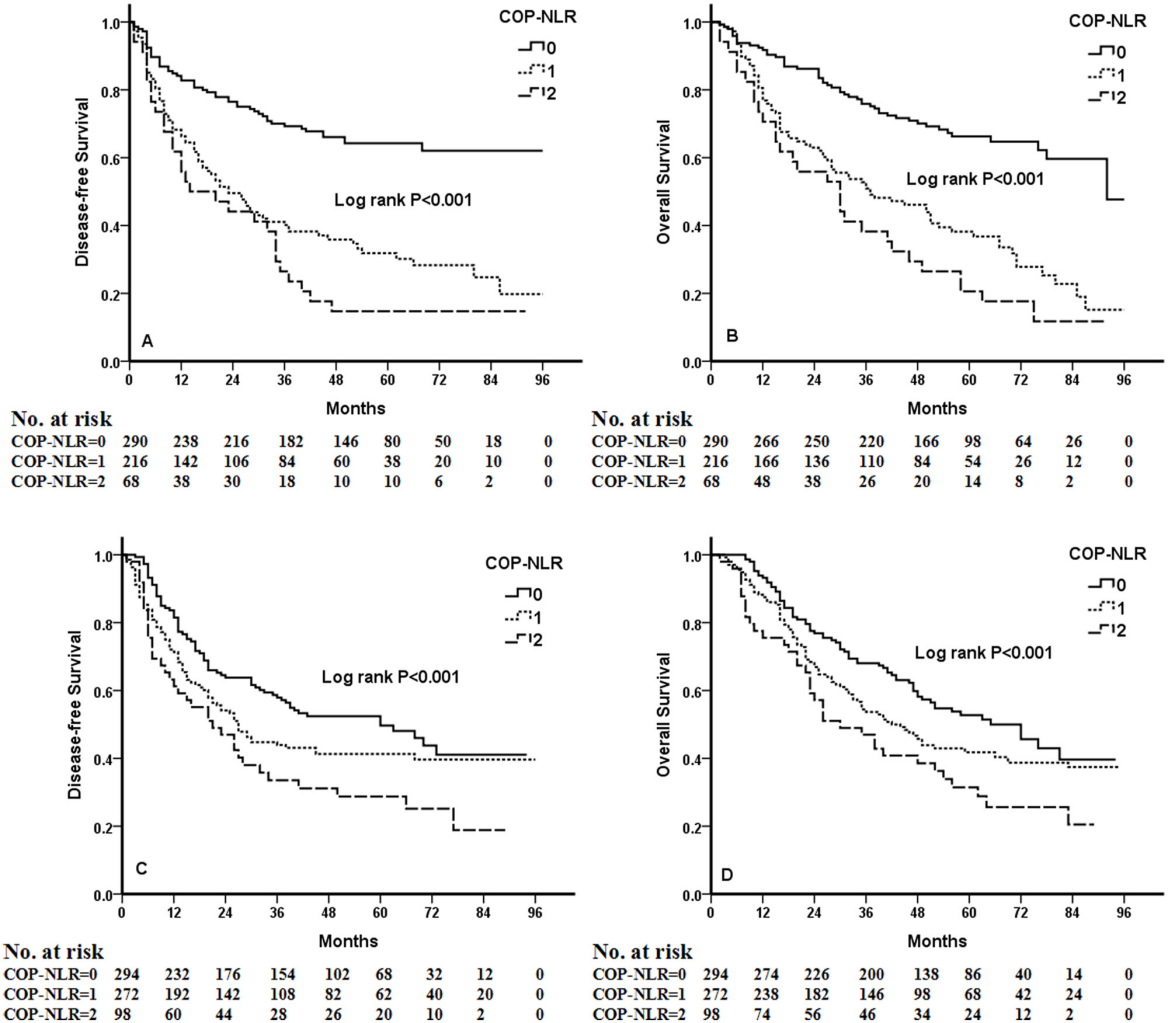
Kaplan-Meier curves for NSCLC patients with different therapies. (A) Kaplan-Meier curve of DFS for surgery alone. (B) Kaplan-Meier curve of OS for surgery alone. (C) Kaplan-Meier curve of DFS for surgery and adjuvant chemotherapy. (D) Kaplan-Meier curve of OS for surgery and adjuvant chemotherapy.

**Fig 5:**
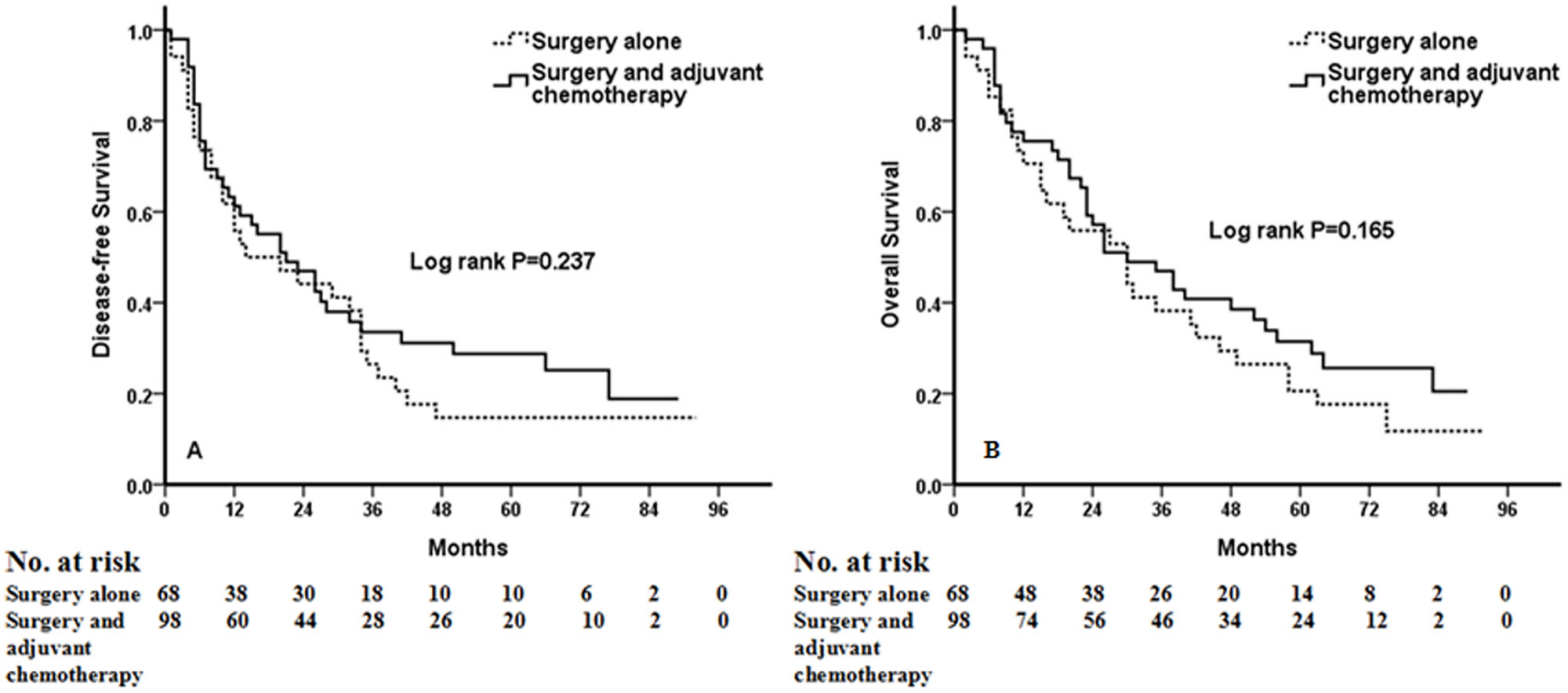
Kaplan-Meier curves for NSCLC patients with COP-NLR = 2. (A) Kaplan-Meier curve of DFS for COP-NLR = 2 NSCLC patients. (B) Kaplan-Meier curve of OS for COP-NLR = 2 NSCLC patients.

Kaplan-Meier analysis and log-rank test demonstrated that there were significant differences in DFS and OS among the three COP-NLR groups (*P*<0.001 and *P*<0.001, respectively) (Fig 1). Patients with COP-NLR = 0 had better prognoses than those with COP-NLR = 1 or COP-NLR = 2. The 5-year survival rate for COP-NLR = 0, COP-NLR = 1, and COP-NLR = 2 was 59.5%, 40.2% and 25.3%, respectively. Thus, the preoperative COP-NLR was able to divide the patients into three independent groups. Similarly, COP-NLR could also separate the patients into three independent groups in SqCC or adenocarcinoma (Fig 2). When the analysis was stratified by stage (I, II, IIIA), we found that DFS and OS were better in the lower COP-NLR group than in the higher COP-NLR group in the stage I, II, and IIIA subgroups (Fig 3). Furthermore, subgroup analyses also revealed that decreased preoperative COP-NLR was significantly associated with better clinical outcomes for patients regardless of whether the patients received adjuvant chemotherapy (Fig 4). To evaluate whether high-risk patients based on COP-NLR = 2 would benefit from adjuvant chemotherapy compared with surgery alone, a Kaplan-Meier analysis and log-rank test were performed. The results of this high-risk subgroup demonstrated no significance difference in DFS and OS (*P* = 0.237 and *P* = 0.165, respectively) (Fig 5).

## Discussion

Immune response within the tumor microenvironment is a critical component of tumor progression and aggressiveness. Understanding these responses has allowed researchers to use immune factors to stratify the prognosis of patients with cancer. Previous studies found that several immune markers could predict the clinical outcome of patients with NSCLC. Using tissue microarray and immunohistochemistry, Suzuki and colleagues found that tumor interleukin-12 receptor β2 (IL-12Rβ2), IL-7R, and stromal FoxP3/CD3 ratio are independent predictors of recurrence in patients with stage I lung adenocarcinoma [14]. Furthermore, in 1290 patients with NSCLC, tumor-infiltrating lymphocytes (mostly CD8+) in the tumor correlated with a better OS [15]. Other immune markers in the tumor microenvironment and peripheral blood of patients with NSCLC have also been reported [16]. In the present study, we investigated the prognostic value of a novel inflammation-based prognostic system, COP-NLR, in 1238 NSCLC patients undergoing complete resection. To the best of our knowledge, this is the first study to show COP-NLR as an independent prognostic factor in patients with NSCLC.

In our study, the prominent point on the ROC curve indicated a cut-off value of 29.0 ×10^4^ mm^-3^ for the platelet count. Although the normal high value for platelet count was 30.0×10^4^ mm^-3^, the cut-off value for reactive thrombocytosis is not clearly defined. Thus, based on the ROC curve, we set 30.0×10^4^ mm^-3^ as the cut-off value of the platelet count. In physiology, platelets have been recognized as mediators of regulatory functions such as immunomodulation, wound healing and angiogenesis [17]. Serving as dynamic reservoirs of various factors, platelets can secrete cytokines and growth factors such as VEGF, platelet-derived growth factor (PDGF), TGF-β and FGF [18–20], which in turn contribute to cancer progression,including angiogenesis, cell migration and proliferation and epithelial to mesenchymal transition [21]. VEGF can promote tumor growth. By inducing the dimerization of EGFR and PDGFR, PDGF results in EGFR transactivation [22]. Platelet-derived TGF-β activates the Smad and NF-kB pathways and promotes cancer metastasis [23]. Cancer cells can also produce IL-6, which has a cell-proliferative effect, triggering the differentiation of megakaryocytes to platelets in the bone marrow. Moreover, platelet receptors and ligands can mediate tumor cell-platelet binding to alter the biological behavior of the tumor [24]. Based on the above results, some studies have investigated the prognostic value of platelet counts in NSCLC and found that platelet counts could predict prognosis [12, 25].

NLR, a measure of the relative difference of between neutrophil and lymphocyte counts, is one index of systemic inflammation. Inflammation contributes to the pathogenesis and progression of lung cancer [26]. The host’s immune response to the cancer is lymphocyte dependent. With a relative lymphocytopenia, patients with a high NLR might have a poorer lymphocyte mediated immune response to cancer, thereby worsening their survival and increasing the potential for cancer progression. Similarly, patients with neutrophilia might have a poor survival. VEGF, a pro-angiogenic factor, the vast majority of which is secreted by neutrophils, plays an important role in cancer progression [27]. The prognostic value of NLR in several types of cancer has been investigated [9, 10, 28–31]. Some studies used the cut-off value of 5.0 [10, 28], whereas other studies used different cut-off values [9, 29–31]. In our study, using ROC curve analysis, we determined that the optimal cut-off value of NLR was 2.3 for predicting prognosis of patients with I-IIIA NSCLC after complete resection.

Hence, both platelet counts and NLR might play an important role in cancer progression. Derived from platelet count and NLR, COP-NLR could expand the predictive value for prognosis of patients with cancer, essentially increasing the unfavorable effect of the platelet count and NLR in cancer progression. Therefore, it is reasonable that there were significant differences among the three COP-NLR groups in tumor-related finding such as age, sex, smoking status, resection type, histological type, and TNM stage. Similarly, it is reasonable that there were significant differences among the three COP-NLR groups in variables such as maximum tumor diameter, WBC count, platelet count, neutrophil ratio, lymphocyte ratio, monocyte ratio, Hb, LDH, albumin, fibrinogen, D-dimer, NLR, and intraoperative blood loss. Low Hb level and hypoalbuminemia are related to poor nutritional status, which reflect cachexia resulting from cancer progression. Cancer cells can release procoagulant molecules and proinflammatory cytokines to activate the coagulation system [32]. As a final degradation product of crossed-link fibrin, D-dimer is a marker of the activation of coagulation and fibrinolysis [33]. As a major acute-phase protein, elevated fibrinogen level is probably induced by an inflammatory response to cancer progression and a prothrombotic state in cancer paitents [34]. Some studies have reported that fibrinogen can be synthesized by cancer cells. However, in our study, platelet count, NLR (data not shown) or COP-NLR was not associated with lymph node metastasis, reflecting the fact that COP-NLR is likely to contribute more to hematogenous metastasis than to lymph node metastasis in NSCLC. In the present study, univariate analysis identified twenty-three variables related to tumor or systemic inflammatory response. Ultimately, fifteen variables were entered in the multivariate analysis, and the results demonstrated that COP-NLR was associated with DFS and OS, along with TNM stage, LDH level and D-dimer. The Kaplan-Meier analysis and log-rank test demonstrated that the preoperative COP-NLR was able to divide the patients into three independent groups. The result is comparable to that of previous studies [11, 12]. Moreover, our study also showed that COP-NLR remained an independent marker in SqCC or adenocarcinoma. When we compared the effect of the COP-NLR in patients with stage I, II and IIIA separately, we found a significant association between COP-NLR level and both DFS and OS in stage I, II and IIIA. At the same time, our results also demonstrated that higher COP-NLR was significantly associated with poor clinical outcome regardless of whether the patients received adjuvant chemotherapy. Furthermore, when the subgroup of patients with high-risk COP-NLR = 2 was analyzed, no benefit of adjuvant chemotherapy could be found.

Over the last decade, numerous systemic inflammation-based prognostic predictors for patients with cancer have been identified. Among these useful and convenient predictors, the Glasgow Prognostic Score (GPS) is regarded as a prognostic milestone [5], and it consists of two acute-phase protein markers, C-reactive protein (CRP) and albumin. Some studies have investigated the prognostic value of GPS in patients with various cancers [5]. However, the clinical utility of GPS might be limited because C-reactive protein is not routinely measured as part of the preoperative examination. Compared with GPS, the preoperative COP-NLR might have a higher applicability for estimation of the systemic inflammation response because proliferation and differentiation of cellular components occurs much faster than protein synthesis when inflammatory cytokines are released. Patients undergoing NSCLC complete resection all undergo preoperative full blood counts,and the platelet count and NLR can be calculated from the data that are already routinely available. Thus, there is no additional expenditure. Moreover, compared to serum tumor markers such as SCC, TPSA, NSE, CA19–9, CEA and Cyfra21–1, hematologic markers are much cheaper and faster.

In summary, based on the results of our study, the preoperative COP-NLR is able to predict the prognosis of patients with NSCLC and divide these patients into three independent groups before surgery, and that high-risk patients base on the preoperative COP-NLR do not benefit from adjuvant chemotherapy.
